# Breastfeeding and the milk resistome shape the establishment and transmission of antibiotic resistance genes in the infant gut microbiome

**DOI:** 10.1080/19490976.2025.2541033

**Published:** 2025-08-04

**Authors:** Zhe Pan, Calum J. Walsh, Conor Feehily, Sai Ravi Chandra Nori, Fionnuala M. McAuliffe, Paul D. Cotter, John MacSharry, Douwe van Sinderen

**Affiliations:** a APC Microbiome Ireland, University College Cork, Cork, Ireland; b School of Microbiology, University College Cork, Cork, Ireland; c The Centre for Pathogen Genomics, Department of Microbiology & Immunology, Peter Doherty Institute for Infection & Immunity, University of Melbourne, Melbourne, Australia; d School of Infection and Immunity, University of Glasgow, Glasgow, UK; e Food Biosciences Department, Teagasc Food Research Centre, Fermoy, Co, Cork, Ireland; f SFI Centre for Research Training in Genomics Data Science, School of Mathematics, Statistics & Applied Mathematics, University of Galway, Galway, Ireland; g UCD Perinatal Research Centre, School of Medicine, University College Dublin, National Maternity Hospital, Dublin, Ireland

**Keywords:** Infant resistome, early resistome, breastfeeding, *Bifidobacterium*, resistome transmission, delivery mode, functional redundancy

## Abstract

The infant resistome, the collection of antimicrobial resistance genes (ARGs) of newborns, is critical for gut microbiota establishment. Using metagenomic sequencing data, we analyzed various 1-week and 1-month postpartum samples to study infant resistome establishment, ARG transmission, and its impact on functional redundancy of the microbiota. A total of 431 samples were analyzed; infant stools (1-week, *n* = 119; 1-month, *n* = 119), maternal stools (1-month postpartum, *n* = 120), and breastmilk (1-month postpartum, *n* = 73). Breastfeeding correlated with increased functional redundancy and altered bacterial-ARG co-occurrence networks in the infant resistome. *Escherichia coli* dominated early resistome dynamics with a higher abundance correlating with reduced functional redundancy. *Bifidobacterium longum* exhibited a consistent negative association with 21 ARGs at one-month in breastfed infants, while four negative relationships between ARGs and *Bifidobacterium bifidum* were observed in formula-fed infants. ARG transmission via breastmilk appears to be gene-specific, with the quinolone resistance gene *sdrM* likely transmitted under maternal antibiotic use. Delivery mode modulated the microbial environment in ways that interact with resistome structure and changing functional redundancy, particularly through genera like *Staphylococcus* and *Streptococcus*. These findings highlight the role of early feeding practices in resistome development and propose functional redundancy as a key ecological framework for understanding infant gut resistome dynamics.

## Background

Early life is a critical window for gut microbiome development, being highly important for the establishment of a balanced microbial ecosystem that impacts host health across its lifespan.^1–4^ Antimicrobial resistance genes (ARGs) can provide a selective advantage for microbes in environments with antibiotic exposure, supporting their persistence and adaptation under such conditions.^5,6^ The collection of gut-associated ARGs in an individual, termed the gut resistome, has gained increased interest since antibiotic resistance poses a potential threat to infants and therefore their short- and long-term health.^7,8^

Recent studies have revealed that establishment of the infant gut resistome is a complex phenomenon, affected by delivery mode, feeding type (i.e. breastfeeding vs formula feeding),^5^ and various other factors (e.g. preterm or term birth^9^). For example, cesarean section-delivered infants harbor a higher relative abundance of ARGs compared to vaginally delivered infants.^10^ The *Enterobacteriaceae* family, which are among the first colonizers of the neonatal gut, are known to frequently carry ARGs due to their inherent adaptability and frequent exposure to selective pressures in various environments.^8,11^ Breastfeeding has been demonstrated to shape the infant gut microbiota composition, being at least partly mediated by human milk oligosaccharides (HMOs), with the consequential dominance of *Bifidobacterium* species which may also help mitigate ARG spread.^5,12,13^ However, how ARGs are distributed across microbial communities, and how *Bifidobacterium* species play a role in limiting ARG spread are questions that cannot yet be answered unequivocally. This requires investigating co-occurrence networks between ARGs and ARG-harboring bacteria, as these patterns provide a deeper understanding of the ecological processes driving the initial establishment, persistence and dissemination of resistomes in microbial communities.

Functional redundancy is an ecological concept, which describes multiple species sharing similar functions/roles in a microbial ecosystem.^14–16^ The highly conserved functional profiles across individuals imply higher functional redundancy, which means members in the community are replaceable in terms of certain functions with a limited impact on the functionality of the overall ecosystem.^14^ A recent analysis of the Earth Microbiome Project dataset revealed that the functional redundancy of polysaccharide-degrading prokaryotic communities enhances the microbial diversity of environmental samples, bridging the ecological definition of community structural variations.^17^ Hence, we speculate that such a concept may also apply to the gut resistome to understand potential mechanisms by which the infant gut resistome establishes itself under different host feeding conditions/delivery modes.

Although breastfeeding fosters neonatal gut microbiota formation and provides nutritional values to infant health, breastmilk is also a source of microbes (*e.g. Staphylococcus* and *Streptococcus*) and is considered to facilitate vertical ARG transmission.^18–20^ However, direct evidence illustrating the transmission of specific ARGs from breastmilk to infants is currently lacking, and it is also unclear to what extent maternal antibiotic usage may increase such transmission events. In the current study, we exploited the metagenomes of a total of 431 fecal samples collected from mother-infant dyads as well as breastmilk samples to study the infant resistome establishment, and to attain ecological insights into patterns of bacteria carrying ARGs. We specifically aimed to shed light on ARG distribution, transfer, and persistence, while we also investigated how breastfeeding influences ARG transmission and whether colonization of (particular) *Bifidobacterium* species mitigates ARG distribution. Finally, by leveraging the concept of functional redundancy, we aimed to determine the contributing bacterial species driving/mitigating ARG dissemination in microbial communities under different feeding conditions and/or associated with distinct delivery modes.

## Materials and methods

### Study cohort and sample collection

As part of the MicrobeMom study (ISRCTN53023014), participants were recruited at the National Maternity Hospital, Dublin, Ireland, between 15^th^ September 2016 and 12^th^ July 2019.^21,22^ Ethical approval was reviewed by the National Maternity Hospital research ethics committee in February 2016 (EC 35.2015). Written informed consent was obtained from all participants, and the study was completed in accordance with the Declaration of Helsinki.

A total of 238 stool samples were collected from infants within 1 week (*n* = 119), and at one-month (*n* = 119) post-partum. A sum of 120 stool samples were collected from mothers at one-month post-partum. Approximately 7 g of stool samples was added to RNA*later* and the remaining sample was stored at −80°C. The RNA*later* was also added to stool-free sample pots and frozen as an environmental control. A total of 73 breastmilk samples were collected from mothers at one-month by pump expression and stored at −80°C.

### Nucleic acid extraction and metagenomic sequencing

The details regarding the nucleic acid extraction and metagenomic sequencing procedures were reported previously.^23^ Briefly, DNA was extracted from all RNA*later* fixed stool samples using AllPrep DNA/RNA kit (Qiagen, Hilden, Germany) following the manufacturer’s instructions. Negative control extraction blanks were included for every 50 samples. Breastmilk samples were centrifuged at 4500 × *g* for 20 min at 4°C. After centrifugation, cream and supernatant were discarded, and pellets were washed twice and resuspended in sterile PBS. DNA was extracted using the MolYsis complete5 kit in accordance with the manufacturer’s instructions. Host cells were lysed by adding a chaotropic buffer, and released nucleic acids were degraded by the MolDNase enzyme. The microbial cells were sedimented and lysed using reagents and proteinase K. Subsequently, microbial DNA was isolated and extracted using spin columns, and 100 μl of DNA was eluted and stored at −20°C. The quantity of DNA was measured using the Qubit™ dsDNA HS Assay Kit and was normalized to 0.2 ng/ml. All libraries were prepared according to the Nextera XT DNA Library Preparation Kit (Illumina) protocol and sequenced using an Illumina NextSeq platform (2 × 300 pair-end).

### Identification of ARGs from metagenomic reads

The raw metagenomic reads were subjected to quality control using Cutadapt (v 2.6)^24^ and FastQC (v 0.11.8).^25^ Trimmed reads were aligned to the human genome using Bowtie2 (v 2.5.1),^26^ aligned reads were removed with Samtools (v 1.17),^27^ and the remainder were converted to quality-controlled microbial FASTQ reads. The ARGs online analysis pipeline (ARGs-OAP v 3.0) was used for resistome analysis.^28,29^ The Structured Antibiotic Resistance Gene (SARG) database identifies ARG types (corresponding to an antibiotic class) and subtypes within each class. Diversities are hence defined as the number of subtypes obtained for each ARG type.^30^ The parameters used for ARG identification were alignment length cutoff of 75 nucleotides, alignment e-value at 1 × 10^−10^, and alignment identity of at least 80%.^30^ ARG abundances were then normalized by the 16S rRNA gene as previously described^28–31^ using the following equation:

$$\mathrm{Abundance}=\overset{n}{\underset{1}{\sum }}\frac{N_{\mathrm{ARG}-\mathrm{Like}\;\mathrm{Sequence}}\times \frac{L_{\mathrm{reads}}}{L_{\mathrm{ARG}\;\mathrm{Reference}\;\mathrm{sequence}}}}{N_{16S\;\mathrm{sequence}}\times \frac{L_{\mathrm{reads}}}{L_{16S\;\mathrm{sequence}}}}.$$

The average abundance (i.e. the mean of the abundances across all samples in the dataset) >0.001 was considered as the detection threshold of antibiotics and ARGs.^32^

### Identification of ARG-encoding bacterial hosts

Metagenomic reads identified as encoding ARGs were first extracted and converted into FASTA format. These reads were then compiled into a multiFASTA file, which served as the reference for aligning quality-controlled metagenomic reads from each sample using Bowtie2.^26^ Subsequently, Samtools (v 1.17)^27^ was used to convert the aligned SAM results to BAM format. Only mapped reads were extracted and sorted. Finally, fastq files that contain reads that were potential genomic carriers of ARGs were generated. These reads were then classified with Kraken2 using the NCBI bacteria database with a confidence score setting to 0.1^33^ and the relative abundance of taxa was calculated at phylum, class, genus, and species level using Bracken.^34^ Networks based on Spearman correlations (*P*_FDR-adjusted_ < 0.05, absolute R^2^ > 0.6) were used to identify co-occurrence networks between the relative abundance of ARG-harboring bacteria and ARG abundance.

### Infant gut ARG profiling

The relative abundance of each antibiotic class and ARG diversities were calculated. Similarities of resistome composition between samples collected at (around) 1-week (1w) and 1-month (1 m) following birth were estimated using Bray-Curtis distance-based Principal Coordinate Analysis (PCoA). The ARG load and the distance to centroid summarized based on PCoA were compared between 1w and 1 m samples, among different feeding types (breastfed, formula, and mixed feeding), antibiotic usage during labor (Yes or No), and delivery mode (Vaginal vs. Cesarian) with Kruskal–Wallis test *P*_FDR-adjusted_ < 0.05. Infants were categorized into three feeding groups: exclusive breastfeeding (1w: *n* = 53, 1 m: *n* = 57), exclusive formula feeding (1w: *n* = 34, 1 m: *n* = 34), and mixed feeding (combination of breastmilk and formula, also named as combined feeding, 1w: *n* = 23, 1 m: *n* = 25). The feeding type was not recorded for nine infants at 1 week and three infants at 1 month. These groupings were used consistently in all analyses. Throughout the manuscript, the term “breastfeeding” or “formula feeding” refers specifically to exclusive breastfeeding or exclusive formula feeding unless otherwise stated.

The ARG load is defined in this study as follows: the total ARG abundance in one sample divided by the number of estimated bacterial cells. The ARG abundance is normalized within the ARGs-OAP pipeline to account for sequencing depth and gene length.^28,31^ Bacterial cell numbers are estimated by correcting 16S rRNA gene counts for known copy number variation across taxa, as previously described.^28,31^ Furthermore, a linear mixed model was used to estimate the fixed effect of these confounding variables (age, feeding type, antibiotic usage in labor, and delivery mode) on the ARG load with family (mother-infant dyad) as the random effect (lmer package in R).^35^ Metadata on antibiotic usage was included where available; however, detailed information on antibiotic type, dose, and duration varied between individuals (Supp metadata). Differentially abundant ARGs were identified between 1w and 1 m using the DESeq2 package in R.^36^

### Evaluation of effects of breastfeeding and delivery mode on the dynamics of functional redundancy of ARG-harboring bacterial community

Functional redundancy of a defined microbial community is considered to be part of the taxonomic diversity (TD) that cannot be explained by the functional diversity (FD), indicating that functional redundancy is calculated as the difference between TD and FD.^37,38^ The Gini-Simpson index (GSI) was chosen as an indicator of TD, and FD was calculated as Rao’s quadratic entropy (Q).^39^ The functional redundancy of ARG-harboring bacteria was calculated using the following equation: functional redundancy = GSI – Q and ranges from 0 to 1. When functional redundancy = 0, it indicates that species are completely different in their functions and when all the species are functionally identical, the functional redundancy of a microbial community tends to be 1.^39^ The functional redundancy of ARG content within ARG-harboring bacteria was calculated using the ‘rao.diversity’ function in the ‘SYNCSA’ package in R,^40^ where functional traits specifically refer to ARG contents. The effects of feeding type (breastfeeding, mixed feeding, and formula feeding) and delivery mode (vaginal vs. cesarean) on functional redundancy were initially compared using the Kruskal – Wallis test (FDR-corrected using the Benjamini – Hochberg method across feeding types and delivery modes). To assess how specific bacterial taxa relate to functional redundancy within each feeding or delivery group, we used linear regression models in which functional redundancy was the dependent variable and log10-transformed read counts of bacterial species of interest (e.g., *E. coli*, *Bifidobacterium*, *Streptococcus*, *Staphylococcus*) served as independent variables. These analyses allowed us to determine whether dominant or functionally relevant species affect redundancy under different early-life exposures. The *P*-values from regression models across bacterial taxa were corrected for multiple testing using FDR.

### Milk ARG profiling

The relative abundance of each antibiotic class and ARG diversities were calculated for milk ARG profiles. The frequency of each antibiotic class was also calculated based on the occurrence of antibiotic classes across all milk samples. The ARG load in milk was calculated using the aforementioned formula and compared to mother and infant ARG load at 1 m (Kruskal–Wallis test *p* < 0.05). The effect of maternal antibiotic usage at 1 m on milk ARG load was further evaluated (Kruskal–Wallis test *p* < 0.05). Subsequently, if and to what extent maternal ARG load affects milk ARG load, and if and to what extent milk ARG load affects infant ARG load were evaluated using linear regression models with maternal antibiotic usage at 1 m as a confounding factor. Whether ARG-harboring bacteria in milk at the phylum, class, genus, and species levels were affected by maternal antibiotic usage at 1 m was analyzed using the Kruskal–Wallis test (*P*_FDR-adjusted_ < 0.05). The similarities of ARG-harboring genus and species under maternal antibiotic usage were evaluated by PCoA.

### Modeling of potential ARG transmission from milk to infant gut at 1 month

The antibiotic class intersections among mother, infant at 1 m, and milk resistome profiles were identified. The DNA sequences of ARGs detected in milk and infant, while not being present in the mother may represent transmitted DNA sequences of ARGs through breastfeeding. Following this, whether ARG abundances in milk affect infant ARG abundances as a result of maternal antibiotic usage was evaluated by the linear regression model.

Furthermore, the transmission of quinolone resistance genes from milk to infant was evaluated. For each of the three quinolone resistance gene subtypes (*norA*, *norB*, *sdrM*), a Bayesian Gaussian regression model was used to estimate the potential for vertical transmission from milk to infant. Predictor variables included ARG abundance in breastmilk and maternal antibiotic usage (Yes/No) at 1-month postpartum. The response variable was the corresponding ARG abundance in the matched infant stool sample. Posterior distributions were used to estimate the relative transmission probability under maternal antibiotic exposure and quantify gene-specific effects.

## Results

### Infant gut ARG profiles and factors affecting resistome establishment

**Figure 1.**
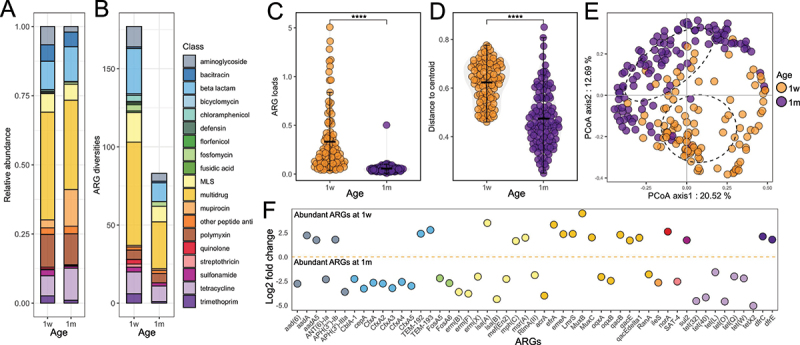
Infant gut ARG profiles and factors affecting resistome establishment.

A total of 177 and 83 ARG subtypes known to confer resistance to 19 and 15 classes of antibiotics were detected in infant stool at 1w and 1 m, respectively. Resistance to bicyclomycin, defensin, florfenicol, and fusidic acid were specific to the infant stool resistome at 1w. A total of 81 ARGs were shown to be shared between 1w and 1 m in the infant stool resistome; however, 96 and 2 (*arnT* and *yojI*) ARGs were specific to 1w and 1 m, respectively. Multidrug resistance ARGs contributed most to the infant stool resistome abundance, of which detected genes were dominant with an average of 0.21 (1 m) and 1.04 (1w) copies per 16S rRNA gene copy and accounted for 32% (1 m) and 39% (1w) of ARG relative abundance in infant stool (Figure 1A). A total of 66 (1w) and 30 (1 m) multidrug resistance ARGs were detected in all infant stool resistomes (Figure 1B). Interestingly, we observed an increased relative abundance of mupirocin resistance genes at 1 m compared to 1w, likely reflecting intrinsic resistance in *Bifidobacterium* species (Figure 1A).

A. The relative abundance of antibiotic classes for infant gut (stool) resistome at 1-week (1w, *n* = 119) and 1 month (1 m, *n* = 119). The colors refer to different classes of antibiotics.

B. The numbers of ARG subtypes belonging to each antibiotic class (ARG diversities) for the infant gut resistome at 1w and 1 m. The colors denote the class of antibiotics to which the gene is predicted to confer resistance.

C. The comparison of ARG load for the infant gut resistome between 1w and 1 m (Kruskal–Wallis test *p* < 0.001****). The colors of each dot refer to infant age (1w or 1 m).

D. Comparison of the distance of points to the centroid on the Principal coordinates analysis (PCoA) plot in relation to infant ages (1w and 1 m, Kruskal–Wallis test *p* < 0.001****). The colors of each dot refer to infant age.

E. The Bray-Curtis distance-based PCoA analysis for the infant gut resistome at 1w and 1 m. The colors of each dot refer to infant ages. The ellipses represent 90% confidential interval.

F. The differential abundant ARGs between 1w and 1 m visualized by log2 fold change (y-axis). Dots above the dashed line represent highly abundant ARGs at 1w, while dots below indicate highly abundant ARGs at 1 m. *n* = 119 for 1w (1-week) and *n* = 119 for 1 m (1-month).

Members of the phyla *Pseudomonadota, Actinomycetota*, *Bacillota*, and *Bacteroidota* were shown to be the main ARG carriers in the infant gut (Fig. S1A). The relative abundance of *Pseudomonadota* and *Bacillota* significantly decreased at 1 m compared to that at 1w, whereas the relative abundance of *Actinomycetota* increased at 1 m, while *Bacteroidota* maintained a similar relative abundance for these two time points (Fig. S1A). At the genus level, *Escherichia* was the most abundant genus carrying ARGs at both 1w (0.62 ± 0.38) and 1 m (0.40 ± 0.29). In addition to *Escherichia*, *Paraburkholderia*, *Enterococcus*, and *Pseudomonas* were also shown to be present as abundant AGR-carrying genera. *Bifidobacterium* (0.31 ± 0.28) was identified to be the second most abundant ARG carrier at 1 m in accordance with the observed increased relative abundance of the gene providing resistance to the mupirocin class, while its abundance at 1w was significantly lower (0.20 ± 0.27, Fig S1B). Only 54 out of 119 (45%) stool samples had detectable *Bifidobacterium* harboring mupirocin-resistance genes at 1w; however, this ARG was detectable for 115 out of 119 (97%) stool samples at 1 m (Fig S1B).

The ARG load was significantly higher at 1w compared to that at 1 m (*p* < 0.01 Figure 1C) and was higher in infants where antibiotic usage in labor was reported compared to those without (*p* < 0.01, Fig. S2A). The ARG compositions were significantly different between 1w and 1 m (Figure 1D, E), although differences were not observed between infants based on reported antibiotic usage during labor (Fig. S2B). A total of 51 differentially abundant ARGs were identified when comparing data from 1w and 1 m fecal samples, among which 20 ARGs were shown to exhibit higher abundance at 1w (Figure 1F). Neither feeding type nor delivery mode appeared to affect ARG load or ARG compositions (Fig. S2C-F). However, interactions among feeding types, delivery modes, and age may affect ARG load (Fig. S2G).

### Escherichia coli as the key species affecting the functionality of the ARG-harboring bacterial community

**Figure 2.**
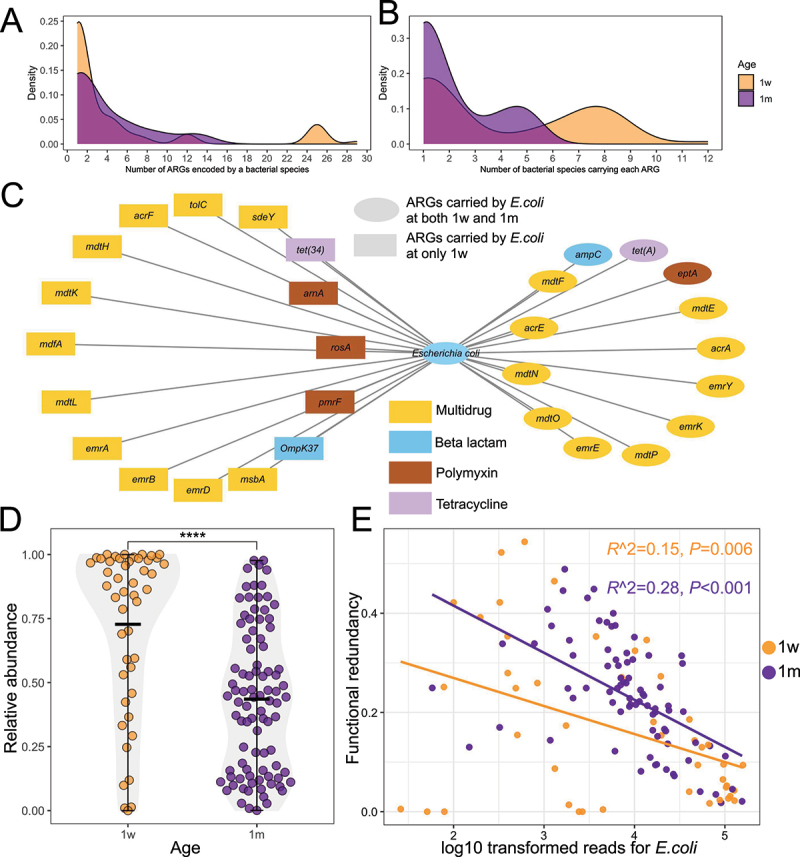
*Escherichia coli* is the key species affecting functionality of the ARG-harboring bacteria community in the infant gut.

ARGs are prevalent in the gut microbiota.^41^ We identified a total of 83 bacterial species that harbored 109 ARGs at 1w in the infant gut. Among bacterial species at 1w, 68 out of 83 (82%) possessed between 1 and 5 ARGs, while the remainder contained more than 5 ARGs (Figure 2A). *Escherichia coli* was identified to possess the highest ARG number, i.e. 29 ARGs, and was detected in 47 out of 119 (39%) infant stool resistomes at 1w. The *Pseudomonas* genus (e.g., *Pseudomonas chlororaphis*) represented the second most diverse ARG carrier with 25 ARGs identified, 17 of which belong to the multidrug resistance class. The *lnuC* from the macrolide-lincosamide-streptogramin class and *mdsB* from the multidrug resistance class were the most frequently identified ARGs, each carried by 12 bacterial hosts at 1w. Of the 109 ARGs identified, 49 (45%) were carried by a single bacterial species, and 58 (53%) were demonstrated to be encoded by fewer than 10 bacterial species at 1w (Figure 2B). At 1 m, 12 bacterial species were predicted to harbor 21 ARGs and 9 of these (75%) species carried a maximum of five ARGs (Figure 2A). *Escherichia coli* was consistently identified as the most diverse carrier of 13 ARGs and was detected in 91 of 119 (76%) infant stool resistomes at 1 m. ARGs represented by *ampC* (associated with beta-lactam resistance), *emrK*, and *emrB* (associated with multidrug resistance) were the most frequently identified ARGs carried by five species from the *Escherichia* genus including *Escherichia coli, Escherichia albertii, Escherichia marmotae, Escherichia sp. E4742* at 1 m. Specifically, 16 ARGs exclusively carried by *E. coli* were detected at 1w, including 11 multidrug resistance genes, 3 polymyxin resistance genes, 1 tetracycline resistance gene, and 1 beta-lactam resistance gene (Figure 2C). In contrast, 13 ARGs, of which 10 are predicted to belong to the multidrug resistance class, were consistently detected in *E. coli* at both 1w and 1 m (Figure 2C).

A. Density plot showing the number of ARGs carried by infant gut bacterial species with color referring to infant age , 1 week (orange, *n* = 119) and 1 month (purple, *n* = 119).

B. Density plot showing the number of bacterial species carrying each ARG, colors referring to infant age.

C. The co-occurrence network between *E.coli* and ARGs. The color of each ARG refers to the class of antibiotic to which the gene is predicted to confer resistance. The square or ellipse shapes refer to ARGs carried by *E.coli*at 1w only or at both 1w and 1 m, respectively

D. The comparison of the relative abundance of *E.coli* in infant gut between 1w and 1 m (Kruskal–Wallis test *p* < 0.001****). The color of each dot refers to infant age.

E. The linear model showing the relationship between log10 transformed reads for *E.coli*and functional redundancy of ARG-harboring bacteria community at 1w (green) and 1 m (gray). *n* = 119 for 1w (1-week) and *n* = 119 for 1 m (1-month).

Furthermore, the relative abundance of *E. coli* was significantly decreased at 1 m compared to 1w (Figure 2D). Interestingly, the functional redundancy of ARG-harboring bacterial communities was significantly higher at 1 m (0.25 ± 0.02) compared to 1w (0.19 ± 0.04, *p* < 0.01). Linear regression analysis further revealed a negative relationship between the log10-transformed relative abundance of *E. coli* and the functional redundancy of ARG-harboring bacterial communities (Figure 2E).

**Figure 3.**
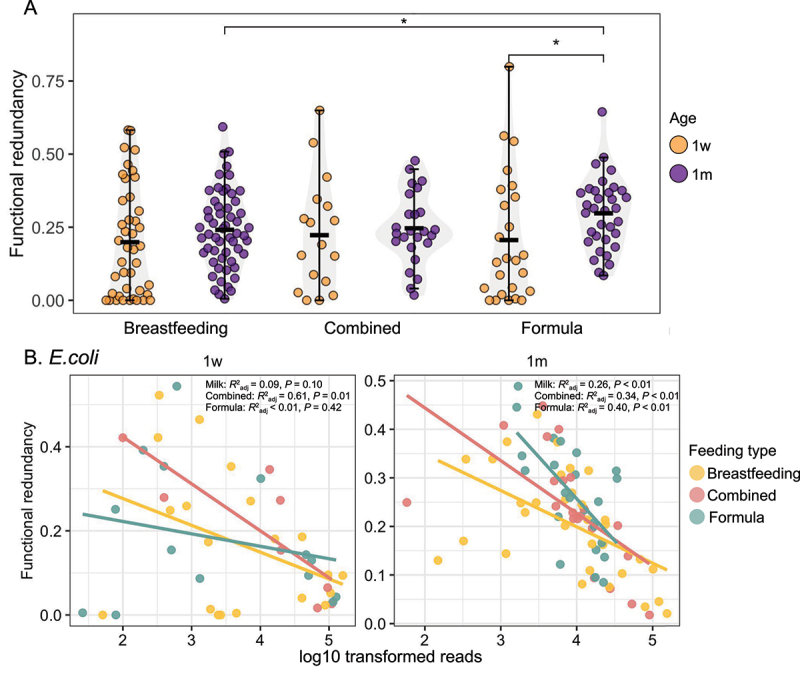
Feeding type relates to the functional redundancy of the ARG-harboring bacteria community of the infant gut.

Although functional redundancy values between 1w and 1 m in breastfeeding and combined feeding infants were comparable, formula-fed infants had a significantly higher functional redundancy at 1 m compared to at 1w (Figure 3A). Notably, mixed-fed infants showed intermediate levels of functional redundancy between breastfed and formula-fed groups, suggesting a potential dose – response relationship to breastmilk exposure, or alternatively, a combined influence of both breastmilk and formula inputs on the gut microbiome and resistome (Figure 3A). When incorporating breastfeeding into functional redundancy analysis, *E. coli* was shown to exhibit a significant negative relationship with functional redundancy in mixed feeding infants at 1w and a trend toward significance in breastfed infants (Figure 3B). At 1 m, *E. coli* was consistently negatively correlated with functional redundancy regardless of feeding type (Figure 3B). In addition, we assessed if key bifidobacterial species including *B. breve*, *B. bifidum*, and *B. longum* affect functional redundancy under different feeding conditions. *B. breve* was shown to exhibit a negative correlation with functional redundancy in mixed-feeding infants only at 1w (Fig. S3A). However, other *Bifidobacterium* species including *B. bifidum* and *B. longum* were not significantly associated with the functional redundancy of ARG-harboring bacterial communities at both 1w and 1 m (Fig S3B-C).

A. Comparisons of the functional redundancy of ARG-harboring bacteria communities in infants with breastfeeding, formula, and combined feeding at 1w and 1 m (Kruskal–Wallis test *p* < 0.05*).

B. The linear model showing the relationship between log10 transformed reads for E. coli and the functional redundancy of ARG-harboring bacteria community under different feeding types at 1w (left panel) and 1 m (right panel). The color of dots and estimated lines refer to different feeding types. Breast feeding *n* = 53 for 1w (1-week) and *n* = 57 for 1 m (1-month), mixed feeding *n* = 23 for 1w and *n* = 25 for 1 m, formula feeding *n* = 34 for 1w and *n* = 34 for 1 m. Feeding type was not recorded for nine infants at 1 week and three infants at 1 month.

### Breastfeeding shapes a varied co-occurrence network between ARGs and bacterial hosts in the infant gut microbiome

**Figure 4.**
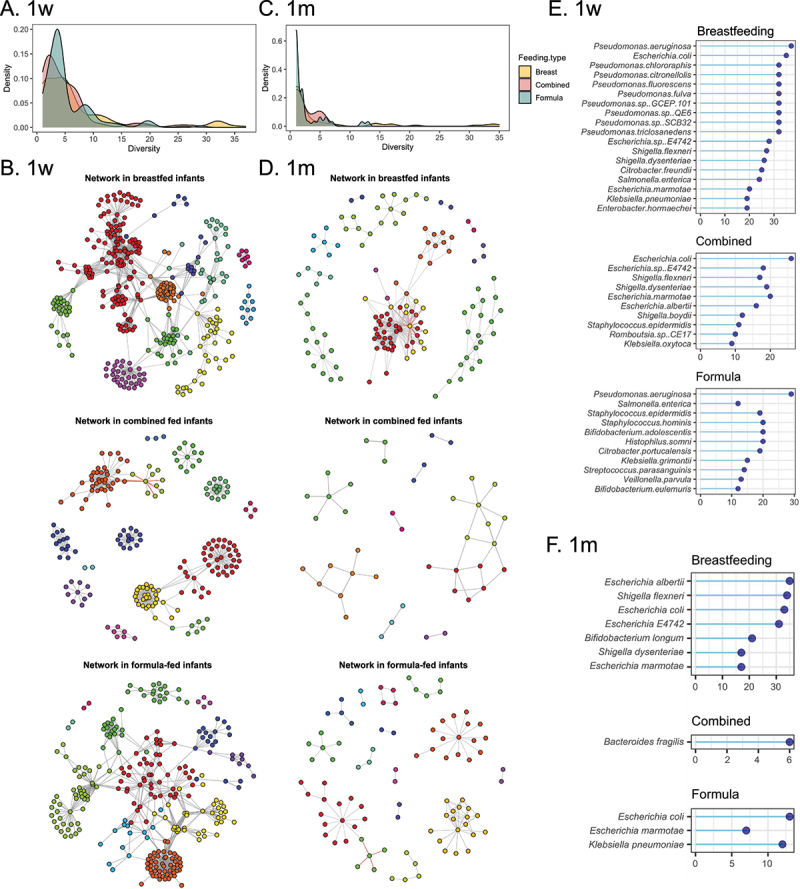
The co-occurrence networks of gut bacterial hosts harboring ARGs in the infant gut microbiome across different ages and feeding type.

Samples were treated as a single dataset for each group (i.e. breastfeeding) at each timepoint (i.e. 1w). At 1w, the gut of infants who were breast-fed, mixed-fed, or formula-fed contained a total of 174, 129, and 113 bacterial species encoding 187, 94, and 140 ARGs, respectively. A larger majority (94 out of 113, 83%) of the assessed formula-fed infant gut microbiota data sets were shown to possess a maximum of five bacterial ARGs when compared to breastfeeding (108 out of 174, 62%) and mixed feeding (81 out of 129, 63%) microbiota data sets (Figure 4A). In breastfed infants, 16 out of 174 (9.1%) bacterial species were found to carry more than 20 ARGs at 1w (Figure 4A). However, at 1w, only 2 out of 113 bacterial species (1.7%; *Escherichia coli*, *Escherichia marmotae*) in mixed-fed infants, and 4 out of 129 (3.1%) in formula-fed infants carried over 20 ARGs (Figure 4A). Studying the co-occurrence networks between bacterial species and ARGs, the modularity (strength of division of a network into clusters, with higher values indicative of co-occurrence networks which tend to be clustered^42,43^) of co-occurrence networks, revealed that values were the highest for mixed-fed infants (value = 0.82) followed by formula-fed infants (value = 0.83) and breastfed infants (value = 0.68, Figure 4B). At 1 month, co-occurrence networks revealed only 62, 17, and 38 bacterial species possessed 71, 26, and 53 ARGs in the gut of infants that had been breast-, mixed- or formula fed, respectively. Among ARG-harboring bacterial species, 49 out of 62 (79%), 16 out of 17 (94%), and 33 out of 38 (87%) bacteria possessed a maximum of 5 ARGs forming the peaks in the density plot in breast-, mixed-, or formula fed infants, respectively (Figure 4C). The modularity of co-occurrence networks in mixed fed (value = 0.75) and formula (value = 0.78) fed infants was higher than that in breastfed infants (value = 0.45, Figure 4D).

A. The density plot showing the number of ARGs carried by gut microbes at 1w among different feeding types (breastfeeding, formula, and combined feeding).

B. The co-occurrence networks of gut bacterial hosts harboring ARGs in breastfed (top), combined (middle), and formula (bottom) fed infants at 1w. The nodes were colored based on the cluster belonging to.

C. The density plot showing the number of ARGs carried by gut microbes at 1 m among different feeding types (breastfeeding, formula, and combined feeding). The colors refer to different feeding types.

D. The co-occurrence networks of gut bacterial hosts harboring ARGs in breastfed (top), combined (middle), and formula (bottom) fed infants at 1 m. The nodes were colored based on the cluster belonging to.

E. Lollipop plots showing the top 10% bacterial hosts carrying ARGs in breastfed, combined, and formula-fed infants at 1w.

F. Lollipop plots showing the top 10% bacterial hosts carrying ARGs in breastfed, combined, and formula-fed infants at 1 m.

Breast feeding *n* = 53 for 1w (1-week) and *n* = 57 for 1 m (1-month), mixed feeding *n* = 23 for 1w and *n* = 25 for 1 m, formula feeding *n* = 34 for 1w and *n* = 34 for 1 m. Feeding type was not recorded for 9 infants at 1 week and 3 infants at 1 month.

Examining the top 10% bacterial species carrying ARGs at 1w, *Pseudomonas aeruginosa* carried the most at 37 and 29 ARGs in breastfed and formula-fed infants, respectively (Figure 4E). However, *E. coli* harboring 26 ARGs was the bacterial species carrying the highest number of ARGs in mixed-fed infants (Figure 4E). Analysis of microbiomes associated with 1 m breastfed infants revealed that *Escherichia albertii* carried the highest number (i.e. 35) of ARGs, followed by *Shigella flexneri*, *E. coli*, and *Escherichia sp. E4742* carrying 34, 33, and 31 ARGs, respectively (Figure 4F). *Bacteroides fragilis* and *E. coli* carrying 6 and 13 ARGs were the most frequently detectable bacterial species carrying ARGs in the mixed- and formula-fed infants at 1 m, respectively (Figure 4F).

### Bifidobacterium longum exhibits the most negative relationship with ARGs in the infant gut

**Figure 5.**
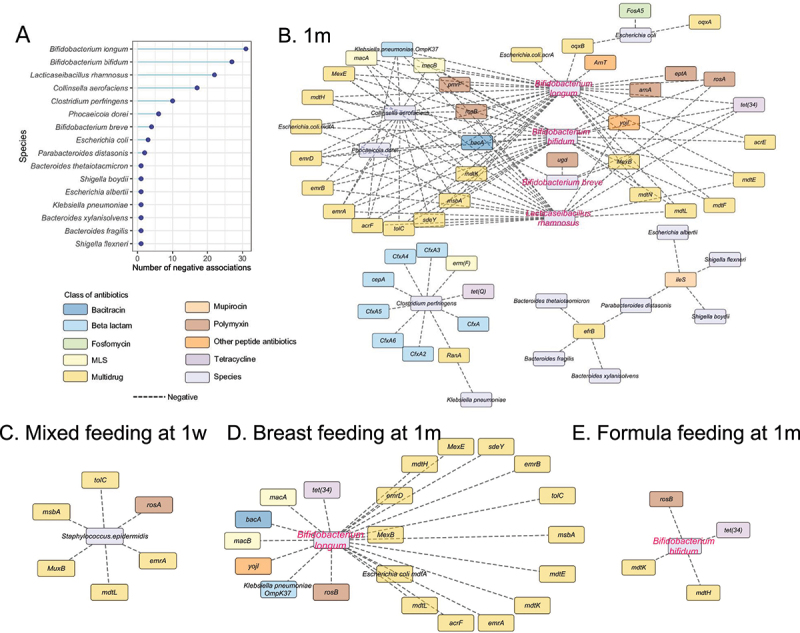
The negative relationships between *Bifidobacterium* and ARGs in the infant gut microbiota.

During the first week following birth, we did not find any significant evidence of a negative relationship between the relative abundance of bacterial species and ARG abundances. At 1 m, a total of 16 bacterial species were shown to exhibit negative relationships with the presence of 46 ARGs, among which *B. longum* was negatively related to 31 ARGs followed by *B. bifidum* and *Lacticaseibacillus rhamnosus* negatively relating to 27 and 22 ARGs, respectively (Figure 5A, B). Among these negative relationships, a total of 22 ARGs were shown to be shared among *B. longum*, *B. bifidum*, and *Lacticaseibacillus rhamnosus* (Figure 5B). Five ARGs (*mdtF*, *mdtN*, and *acrE*, which are associated with multidrug resistance, and *pmrF*, and *arnA*, which are associated with polymyxin resistance) were shown to be negatively associated with the abundance of both *B. longum* and *B. bifidum* (Figure 5B). The *acrA* and *oqxB* (multidrug resistance), *arnT* (resistance to peptide antibiotics), and *eptA* (associated with polymyxin resistance) were only negatively associated with *B. longum* (Figure 5B). *B. breve* was found to be only negatively related to four ARGs including *bacA* (bacitracin resistance), *mexB* (multidrug resistance), *ugd* (polymyxin resistance), and *tet(34)* (tetracycline resistance) (Figure 5A, B).

A. Bacterial hosts that exhibit the most negative relationship with ARGs at 1 m.

B. The co-occurrence networks showing the negative relationships between Bifidobacterium and other species and ARGs at 1 m. The color of each ARG refers to the corresponding class of antibiotics.

C. The co-occurrence networks showing the negative relationships between Staphylococcus epidermidis and ARGs observed in mixed feeding infants at 1w. The colors refer to different classes of antibiotics.

D. The co-occurrence networks showing the negative relationships between Bifidobacterium longum and ARGs in breastfed infants at 1 m. The colors refer to different classes of antibiotics.

E. The co-occurrence networks showing the negative relationships between Bifidobacterium bifidum and ARGs in formula-fed infants at 1 m. The colors refer to different classes of antibiotics.

Furthermore, by incorporating breastfeeding effects into co-occurrence networks between bacterial species and ARGs, we observed that the presence of *Staphylococcus epidermidis* negatively related to *tolC, msbA, MuxB, mdtL, emrA* as well as *rosA* in mixed fed infants at 1w (Figure 5C). At 1 m, *B. longum* was shown to exhibit a negative relationship with 21 ARGs, most of which were associated with multidrug resistance (14 out of 21, 67%) in breastfed infants (Figure 5D); however, a negative relationship between *B. bifidum* and 4 ARGs was observed in formula fed infants (Figure 5E).

### The Streptococcus and Staphylococcus genera link delivery modes to the functional redundancy of ARG-harboring communities

**Figure 6.**
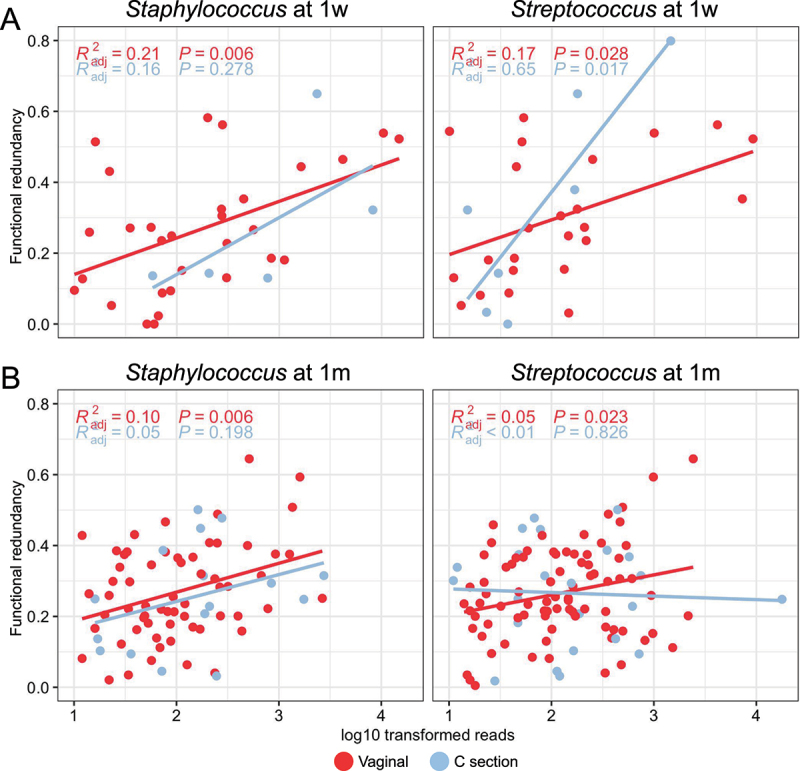
Relations between log10 transformed reads of *Staphylococcus* (left panel) and *streptococcus* (right panel) genus and the functional redundancy of ARG-harboring communities at 1w (A) and 1 m (B) in infants relative to different delivery modes. The color of each dot refers to different delivery modes: vaginal (red, *n* = 94), C-section (blue, *n* = 25).

Although delivery mode does not appear to significantly affect functional redundancy of the infant resistome at either 1w or 1 m (*p* > 0.05), the abundance of *Streptococcus* genus exhibited a strong positive relationship with functional redundancy in infants born by cesarean section at 1w (Figure 6A). Nonetheless, such a relationship was not observed at 1 m (Figure 6B). However, the abundance of *Staphylococcus* and *Streptococcus* was shown to elicit a positive relationship with functional redundancy for vaginally delivered infants at both 1w, in particular, and 1 m though the explanatory power is low (all *p* < 0.05, *R*^*2*^ ranging from 0.05 to 0.21, Figure 6A, B). At species level, *Streptococcus mitis*, which was only identified in vaginally delivered infants, was shown to exhibit a positive relationship with functional redundancy at 1w (Fig S4A). For the *Staphylococcus* genus, two common ARG-harboring species, *Staphylococcus epidermidis*, and *Staphylococcus aureus*, showed no significant relationship with the functional redundancy of ARG-harboring communities (Fig S4B-C).

### Milk resistome profiles and relationships with maternal antibiotic usage and infant ARG load

**Figure 7.**
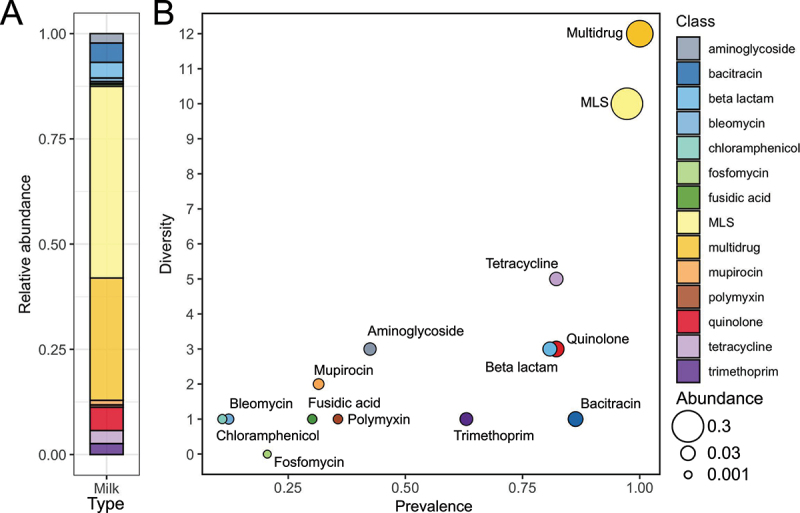
ARG profiles present in human breastmilk at one-month post-partum.

A total of 44 ARGs conferring resistance to 14 classes of antibiotics were identified in the milk resistome at 1 m (*n* = 73). Among identified antibiotic classes, resistance to macrolide-lincosamide-streptogramin (MLS) was shown to be the most abundant (45%), with 10 subtypes and 97% occurrence in milk samples, followed by multidrug resistance with 12 subtypes comprising 29% of resistome abundance detected in all milk samples (Figure 7A, B). There were five tetracycline, three quinolone, three beta-lactam, and three aminoglycoside subtypes detected in the milk resistome and they only accounted for an average of 3.0%, 5.0%, 3.7%, and 2.2% milk resistome abundances, respectively (Figure 7A).

Breast feeding *n* = 53 for 1w (1-week) and *n* = 57 for 1 m (1-month), mixed feeding *n* = 23 for 1w and *n* = 25 for 1 m, formula feeding *n* = 34 for 1w and *n* = 34 for 1 m. Feeding type was not recorded for nine infants at 1 week and three infants at 1 month. The relative abundance of antibiotic classes of the milk resistome at 1-month post-partum, the colors refer to different class of antibiotics.

B. The scatter plot showing the frequency and diversity of antibiotic classes. The prevalence (x-axis) refers to the number of milk samples containing a ARG predicted to encode resistance to each class of antibiotic, and the diversity refers to the number of ARG subtypes belonging to each antibiotic class. The size of each dot is proportional to the abundance of each antibiotic class. *n* = 73 breastmilk samples.

Compared to the ARG load in maternal (0.001 ± 0.002) and infant (0.01 ± 0.06) resistomes (as based on fecal sample analyses) at 1 m, the ARG load in the milk resistome was significantly higher (0.07 ± 0.09, Fig. S5A). When incorporating maternal antibiotic usage at 1 m, ARG load in the maternal resistome was higher in mothers treated with antibiotics compared to those who were not (Fig. S5B). However, maternal antibiotic usage at 1 m did not affect ARG load in milk regardless of maternal antibiotic usage at 1 m (Fig. S5C, D). Likewise, ARG load in milk was less predictive for ARG load in infants at 1 m (Fig. S5E).

At phylum level, ARG-harboring bacteria in mother’s milk are mainly dominated by *Bacillota* (67 out of 73; 92%) and *Pseudomonadota* (6 out of 73; 8%) with *Staphylococcaceae* and *Xanthomonadaceae* being the most abundant families, respectively (Fig. S6A). At the genus level, *Staphylococcus* and *Streptococcus* were the most abundant ARG-harboring genera, followed by *Acinetobacter* (Fig. S6B). The relative abundance of *Bacillota* phyla was higher in milk collected from the mother with antibiotic treatments at 1 m, while *Pseudomonadota* levels were unchanged (Fig. S6C). Neither the *Staphylococcus* nor *Streptococcus* genus was shown to exhibit differential abundance in milk samples collected from the mother with/without antibiotic treatments at 1 m (Fig. S6D). Likewise, bacterial compositions at both genus and species levels were not separated by maternal antibiotic usage at 1 m (Fig. S6E).

### Potential transmission of milk quinolone resistance genes to the infant gut resistome

**Figure 8.**
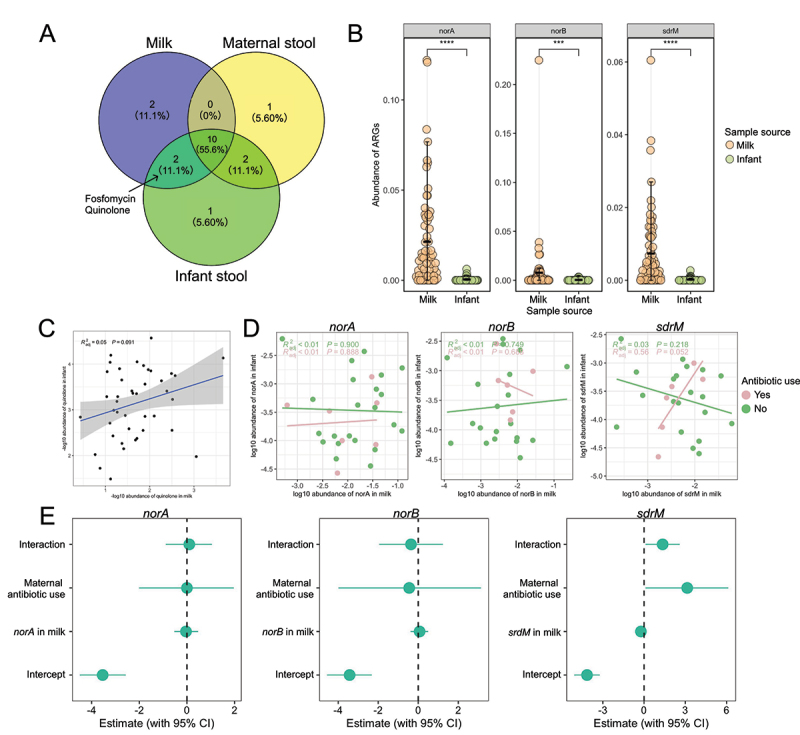
The transmission of quinolone subtypes from maternal breastmilk to the infant gut microbiota.

A total of 10 ARG classes were all detected in milk, maternal, and infant resistome at 1 m. Resistance genes for two antibiotic classes, quinolone and fosfomycin, were only detected in the milk and infant resistome, indicating the potential transmission of these ARGs from milk to infant (Figure 8A). Since genes encoding fosfomycin was detected in only 20% of assessed milk samples and accounted for 0.1% of the total milk resistome (Figure 7A, B), only quinolone (detected in 82% milk samples and accounted for an average of 5.0% milk resistome) was included to study the effect of breastfeeding in ARG transmission. A total of 66 infant stool samples were compared to their corresponding breastmilk samples. Three subtypes *norA, norB*, and *sdrM* were detected in 29, 26, and 26 milk samples, respectively, as well as corresponding infant stool samples (Table S1). All three subtypes were shown to exhibit a significantly higher abundance in milk compared to those in the infant stool resistome (Figure 8B). Only 5% of total quinolone abundances in infant stool resistome were explained by the abundance of quinolone in the milk resistome with a tendency toward significance (*p* = 0.091, Figure 8C). However, by incorporating quinolone subtypes and maternal antibiotic usage at 1 m, we identified that the abundance of the *sdrM* gene in the infant stool resistome was significantly positively related to *sdrM* abundance in milk collected from mothers treated with antibiotics (Figure 8D).

A. Venn diagram showing the shared class of antibiotics among milk, infant, and maternal stool.

B. The differences in the abundance of quinolone subtypes (norA, norB, and sdrM) in milk and infant stool (Kruskal–Wallis test *p* < 0.001****). Colors indicate sample origin, milk (blue), maternal stool (yellow), infant stool (green).

C. Linear regression model showing the relationship between the abundance of quinolone resistance genes in milk and infant stool (All abundance values were -log10 transformed).

D. The linear regression model showing the relationship between the log10 transformed abundance of quinolone resistance genes (norA, norB, and sdrM) in milk collected from mothers with/without antibiotic treatments and the log10 transformed abundance of quinolone resistance genes in infant stool. Colors indicate whether the mother had received antibiotics at 1-month postpartum, Yes (pink, *n* = 7 for norA, *n* = 6 for norB, *n* = 6 for sdrM), No (green, *n* = 22 for norA, *n* = 20 for norB, *n* = 6 for sdrM). Note that not all milk samples contained all three quinolone subtypes, resulting in different sample sizes across the models.

To further assess the likelihood of gene-specific transmission under maternal antibiotic exposure, we applied a Bayesian Gaussian model for each of the three quinolone resistance gene subtypes (*norA*, *norB*, and *sdrM*), incorporating milk ARG abundance and maternal antibiotic usage at 1-month postpartum as predictor variables. Of the three, the Bayesian Gaussian model revealed that the transmission possibility of *sdrM* from milk to infant is 23.12 (e^3.14^) times higher under the maternal antibiotic usage condition (Figure 8E), supporting the hypothesis of enhanced vertical ARG transfer under antibiotic pressure. Neither *norA* nor *norB* abundances in infant stool resistome were related to corresponding ARG abundances in the milk resistome and did not support the potential transmission from milk to infant regardless of maternal antibiotic usage (Figure 8D, E).

Furthermore, *S. aureus* was the microbial species carrying the most diverse set of ARGs and was predicted to carry quinolone resistance genes including *norA, norB*, and *sdrM* in milk. It was also predicted to carry ARGs associated with resistance to beta-lactam (*blaz, mecA*), fusidic acid (*fusB*), multidrug (other major facilitator superfamily transporter, *LmrS*), tetracycline (*tet38*), and trimethoprim (*drfC*) (Fig. S7A). Interestingly, the *norA* gene was also carried by *S. epidermidis*, which was the most abundant ARG-harboring bacterial species in milk (Fig S7B). ARGs including *mupA*, *tetA*, *lnuA*, and *APH (3’)-lb* were also carried by multiple bacteria (Fig. S7A). ARGs including *patA, patB* and *pmrA* were only harbored by the *Streptococcus* genus (Fig. S7A). Similar to the observation in the milk resistome, and the infant resistome, all three quinolone ARGs (*norA, norB*, and *sdrM*) were harbored by *S. aureus*. The *norA* was also carried by *S. epidermidis, Staphylococcus hominis*, and *Streptococcus sp.116-D4* (Fig S8). The *norB* and *sdrM* were carried by *Staphylococcus sp. SN-1* and *S. epidermidis*, respectively (Fig S8).

## Discussion

Our study comprehensively assessed the presence, diversity, and frequency of infant gut ARGs, from which certain observations regarding resistome establishment and transmission dynamics were derived in the infant gut during the first month of life. By integrating metagenomic sequencing from mother-infant dyads and breastmilk samples, we elucidated the complex interplay between feeding practices and the presence of specific microbial species in shifting ARG compositions over time and maintaining gut microbial ecosystem stability. This is characterized by the diversification of ARG-harboring bacterial taxa (*i.e*. increased relative abundance and frequency of *Bifidobacterium* genus) and altered functional redundancy in the gut microbiome. We also note that breastmilk is the potential resource for the vertical transmission of ARGs to the infant gut as evidenced by the uniquely acquired ARGs in the infant gut microbiome.

We highlight that the infant gut microbiome and resistome are shaped by multiple factors,^5,44,45^ and in particular breastfeeding was associated with differences in bacterial – ARG interactions and gut resistome profiles in our cohort. Partially in accordance with previous studies which reported tetracycline resistance genes as the most abundant ARG in the infant gut,^8,10^ we found that our cohort was represented by a higher relative abundance of multidrug-resistance genes and a higher prevalence of tetracycline resistance genes (detected in more than 90% infant stools). This discrepancy may be due to the differences in study populations (the HELMi cohort collected infant stool samples from 3 to 24 months of life^10^ vs. the MicrobeMom cohort collected infant stool samples during the first week and first month of life in our study) or regional antibiotic prescribing patterns. Although some ARGs matched pathogen genomes (e.g., *Shigella flexneri*), the presence of such an ARG does not confirm its transcriptional activity or contribution to resistance under *in vivo* conditions. Due to the low microbial biomass in breastmilk and small sample volumes collected from mother-infant dyads, RNA-based profiling (i.e. metatranscriptomic) was not technically feasible in our study. Future work confirming the functional roles of key ARGs will benefit from using higher-input samples or isolate-based testing.

During the first week of life, we observed *E. coli* as the major carrier of ARGs in accordance with previous findings.^8,46,47^ We further applied the ecological concept of functional redundancy to understand how *E. coli* abundance may affect infant gut ARG profiles through the variation of gut microbial ecosystem stability. Functional redundancy, in brief, describes the ability of various taxonomically differing microorganisms to contribute to an ecosystem in similar ways through redundant functional potential.^48^ Our study revealed that higher abundance of *E. coli* in the early gut microbiome correlates to reduced functional redundancy during the initial colonization phase (first week following birth). This negative correlation may be explained by the ecological role of *E. coli* as an early colonizer that rapidly dominates the gut environment.^49^ *E. coli* may outcompete other bacteria for resources, reducing microbial diversity^43,50,51^ and thereby restricting the distribution of ARGs across multiple taxa. Additionally, the strong ability to acquire (horizontal gene transfer, HGT) and maintain ARGs^52^ may decrease the necessity for functional redundancy, as resistance genes are primarily concentrated within a single dominant species rather than distributed among a broader range of bacterial taxa. As microbial diversity in the infant gut increases over time within the same cohort,^23^ ARGs may become distributed across a broader range of bacterial species, contributing to a redundant community. This process may facilitate ARG distribution in the gut ecosystem while reducing the dominance of a single carrier. To our knowledge, this is the first study to apply the ecological concept of functional redundancy to the infant gut resistome. Prior work has shown that functional redundancy supports stable metabolic output (i.e. maintenance of consistent biochemical outputs even as species composition changes^48^) and resistance to dysbiosis in the adult gut (compensate for the loss or overgrowth of specific bacteria and support long-term microbial stability of a given ecosystem^14^). By extending this concept to ARG-harboring bacteria, we propose that functional redundancy may help to maintain a more balanced ARG-harboring bacterial community during early microbial succession.

Our results indicate that by 1 month of age, functional redundancy increases, supporting the potential redistribution of ARGs among diverse taxa. It is also possible that the observed increase in bacterial species negatively associated with ARGs at 1 m is partly due to the higher microbial diversity at 1 m where an expanded microbial community reduces the relative abundance of ARG-encoding species, even if their absolute numbers remain constant. This dynamic may be essential for understanding how ARGs persist within the gut microbiome, even in the absence of strong antibiotic selection pressures.

It is noted that the functional redundancy values in breastfed infants at 1w and 1 m were comparable, which suggests that breastfeeding is associated with a resilient microbial ecosystem that may limit ARG dissemination. Breastfeeding selectively promotes colonization of beneficial bacteria such as *Bifidobacterium*,^53,54^ which may facilitate a balanced microbial ecosystem where ARGs are less likely to be concentrated within specific taxa, reducing the potential for resistance gene dissemination. In accordance with this, co-occurrence networks in breastfed infants were shown to be less modular at both 1w and 1 m, suggesting greater microbial connectivity within the early life resistome and a more interconnected microbial ecosystem. As early colonizers of the neonatal gut, *Bifidobacterium* species efficiently metabolize HMOs derived from breastmilk, conferring a selective advantage in breastfed infants and promoting a gut microbiome composition that favors beneficial bacteria over opportunistic pathogens.^12,53,55–57^ Indeed, *B. longum* was shown to elicit a strong negative association with 21 ARGs at 1 m in breastfed infants, suggesting its potential role in mitigating ARG dissemination in the context of breastfeeding.

Instead, formula-fed infants showed a reduced number of ARG-negative associations, limited to *B. bifidum* and four ARGs. The functional redundancy was increased in formula-fed infants at 1 m, which may reflect an adaptive response to nutritional differences between formula and breastmilk. It is noted that the co-occurrence network in the mixed-feeding group showed the highest modularity, which indicates the presence of distinct bacterial – ARG clusters derived from both breastmilk and formula-associated microbiota. Such modular structures may reflect functional compartmentalization, potentially promoting system resilience,^58,59^ yet also increasing the potential for localized ARG enrichment. These findings support the idea that mixed feeding introduces a more complex ecological structure to the infant gut resistome. Further investigations are needed to determine whether these differences have long-term implications for microbiome stability and ARG dissemination in early life.

Although breastmilk contains a diverse resistome, its direct impact on the infant gut resistome appears limited. Breastmilk was shown to exhibit a high abundance of MLS resistance genes and a high overall ARG load, although these did not directly correlate with ARG load and prevalence in infants, suggesting that most maternally derived ARGs do not persist effectively into the neonatal gut microbiome. However, specific concerns remain regarding the transmission of quinolone resistance genes, particularly *sdrM*. This ARG was uniquely observed in milk and infant resistome (except maternal stool resistome) in our study. Maternal antibiotic use further raises concerns about milk-mediated ARG transmission, as it significantly increased the likelihood of *sdrM* transfer from breastmilk to the infant gut microbiome, with Bayesian modeling indicating a 23-fold higher transmission probability in antibiotic-exposed mothers. This highlights the necessity of evaluating antibiotic usage during lactation and its role in shaping neonatal resistome. While our data suggests sharing of ARGs, evidence of direct transmission and the possibility of a common environmental or external reservoir from which both mother and infant had acquired the ARG require further investigation.

Moreover, members of the genus *Staphylococcus* (i.e. *S. aureus*, *S. epidermidis*) were found to be the major carriers of *sdrM* in both breastmilk and infant gut microbiomes. Given that *S. aureus* can be transmitted between healthy, lactating mothers and their infants by breastfeeding,^60^ its presence in breastmilk may serve a functional role within the maternal-infant microbial ecosystem. All mothers undergoing cesarean section receive intravenous prophylactic antibiotics to reduce the chance of postpartum infection and this may explain some of these findings. While the presence of potential pathogens such as *Pseudomonas* and *Staphylococcaceae* in breastmilk may have beneficial effects in the infant’s immune system development,^61^ this would require further studies of the infant immune cytokine levels but also maternal milk-derived antibodies to determine the potential effects on the microbiota. Indeed, a recent study has revealed maternal immunoglobulin type (IgM, IgG, IgA2) and specific microbe binding may also play a role in microbiota persistence in the developing gut revealing another layer of microbiota regulation, which could be further investigated in terms of functional redundancy.^62^

Interestingly, the association between *Staphylococcus* and functional redundancy was more significant in vaginally delivered infants, suggesting that the infant gut microbial environment shaped by vaginal delivery enhances bacterial interactions and ARG distribution across multiple taxa. Vaginal delivery exposes infants to a more diverse microbial community, including *Bifidobacterium* and *Bacteroides*,^63^ which may promote a more functionally redundant and interconnected microbial network. In this diverse ecosystem, *Staphylococcus* may act as a transient colonizer that interacts with multiple taxa, thereby contributing to functional redundancy. The low explanatory power suggests that such relationships between *Staphylococcus* and functional redundancy may be confounded by other variables, such as host immunity, and environmental exposures, however, future explorations are needed.

Our findings highlight the complexity of microbial succession, functional redundancy, and ARG dissemination in the infant gut and are the first to provide ecological insights for factors such as breastfeeding and delivery mode in relation to the composition and stability of the infant gut resistome during the first month following birth. This work is the first to integrate the ecological theory of functional redundancy into resistome analysis, offering new insight into how resistance gene compositions may be distributed and stabilized during early gut colonization. Breastfeeding relates to a more stable microbial ecosystem, while formula feeding relates to the stability of the infant gut microbiome through the changing of the functional redundancy and reduced negative relationships between *Bifidobacterium* and ARGs. We also obtained evidence that breastfeeding with maternal antibiotic exposure increases the transmission possibility of certain ARGs from breastmilk to the infant gut. The observed associations between *Staphylococcus* and functional redundancy further highlight that delivery mode shapes ARG distribution in early life, reinforcing the need to investigate ecological drivers of resistome persistence and evolution. It is noted that this study is observational in design, the relationships we report are associative rather than causal. Further experimental or longitudinal intervention studies are needed to confirm causality. While our analyses accounted for antibiotic usage during labor and at 1-month postpartum, it is important to note that detailed metadata on antibiotic exposure was not uniformly available across all participants. We highlight this as a limitation of our study, and we recommend that future research include more comprehensive pharmacological metadata to better elucidate relationships between specific antibiotic exposure and functional redundancy of gut microbiome. Taken together, feeding practices play a pivotal role in shaping the infant gut resistome and the succession of ARG-harboring bacterial communities, with potential implications for antimicrobial resistance mitigation strategies.

## Supplementary Material

Pan et al_Supp metadata.xlsx

Pan et al_Supp Figures.pdf

Pan et al_Supp Table.docx

## Data Availability

All raw sequencing data used in this study is available in the ENA repository under accession number PRJEB48251.
