# Supplementary material for: Breastfeeding and the milk resistome shape the establishment and transmission of antibiotic resistance genes in the infant gut microbiome
Source: Gut Microbes. 2025 Aug 4;17(1):2541033. doi: 10.1080/19490976.2025.2541033 (PMC12323436; doi:10.1080/19490976.2025.2541033)
Supplement: Pan et al_Supp Table.docx [file KGMI_A_2541033_SM3864.docx]

| ARGs from quinolone class | Detected in breastmilk | Detected in corresponding infant stool samples at 1m | Numbers |
| --- | --- | --- | --- |
| *norA* | √ | √ | 29 |
|  | × | √ | 7 |
|  | √ | × | 25 |
|  | × | × | 5 |
| *norB* | √ | √ | 26 |
|  | × | √ | 14 |
|  | √ | × | 17 |
|  | × | × | 9 |
| *sdrM* | √ | √ | 26 |
|  | × | √ | 8 |
|  | √ | × | 23 |
|  | × | × | 9 |

Table S1. The summary of antibiotic resistance genes from the quinolone class in breastmilk and infant stool samples at 1m. The ‘√’ refers to the detectable ARGs in samples, and ‘×’ means ARGs were not detected in samples.
