# Supplementary material for: Breastfeeding and the milk resistome shape the establishment and transmission of antibiotic resistance genes in the infant gut microbiome
Source: Gut Microbes. 2025 Aug 4;17(1):2541033. doi: 10.1080/19490976.2025.2541033 (PMC12323436; doi:10.1080/19490976.2025.2541033)
Supplement: Pan et al_Supp Figures.pdf [file KGMI_A_2541033_SM3865.pdf]

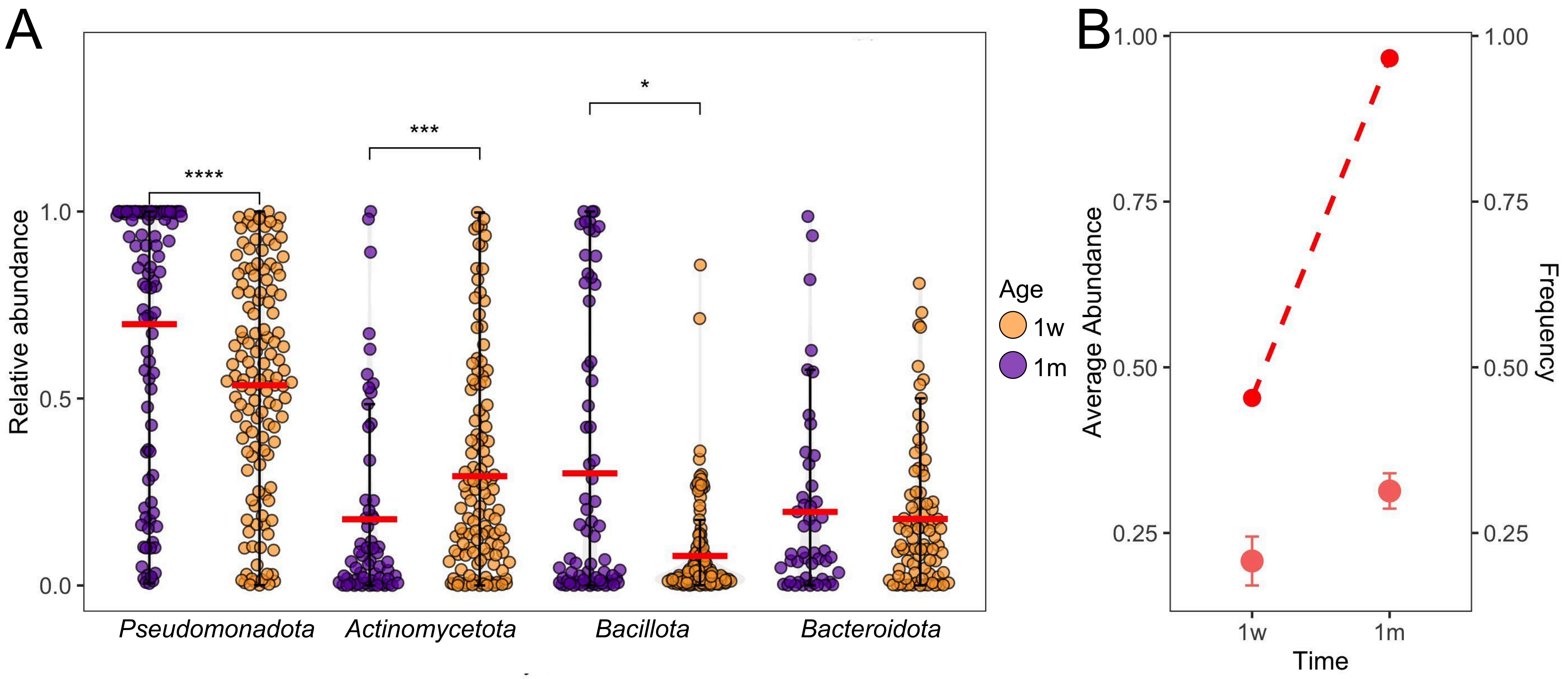

A
Relative abundance
1.0
0.5
0.0
****
***
*
Pseudomonadota
Actinomycetota
Bacillota
Bacteroidota
Age
1w
1m
B
Average Abundance
Frequency
1.00
0.75
0.50
0.25
1w
1m
Time

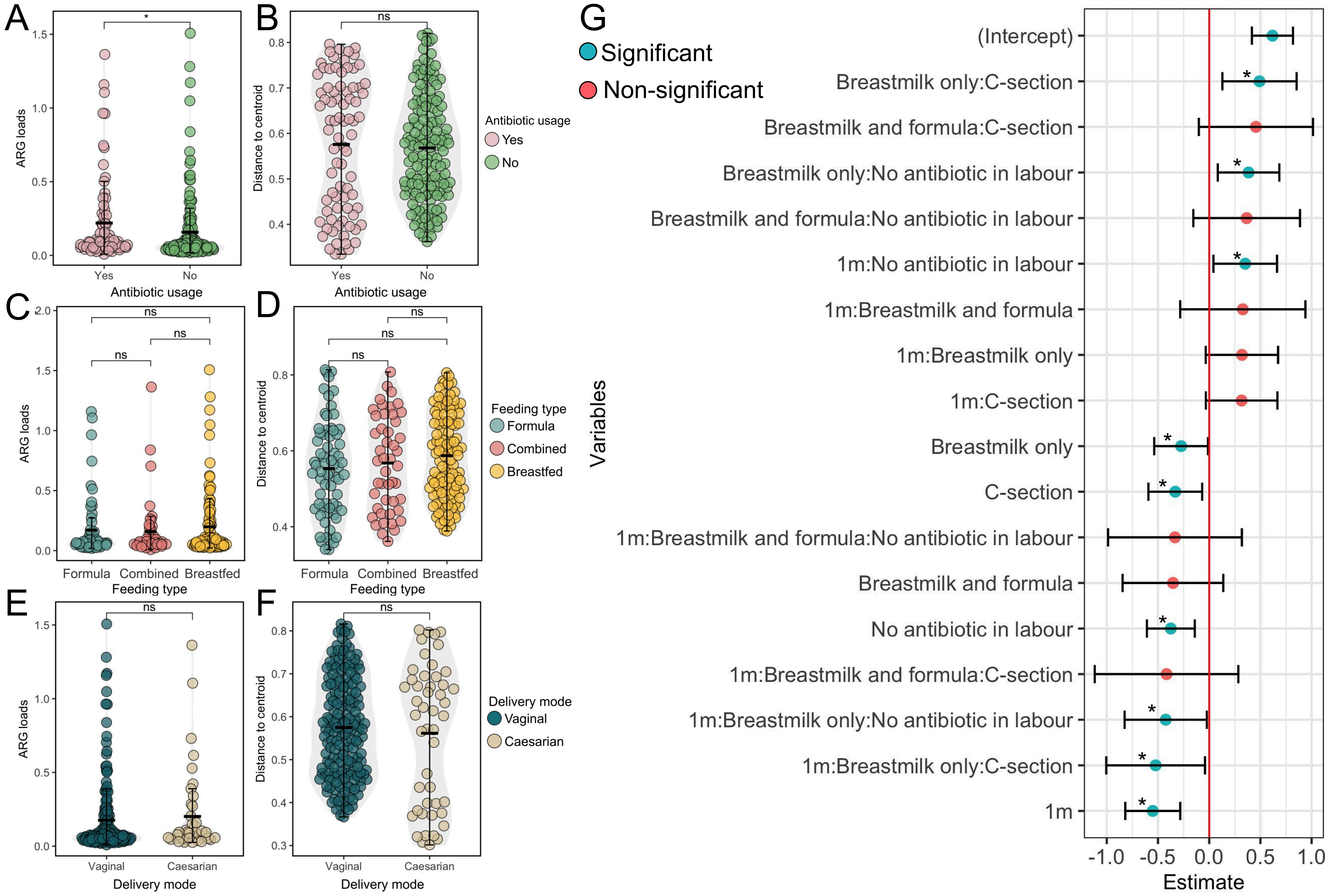

A
B
C
D
E
F
G
ARG loads
Distance to centroid
Antibiotic usage
Yes
No
ns
*
Feeding type
Formula
Combined
Breastfed
Delivery mode
Vaginal
Caesarian
Significant
Non-significant
(Intercept)
Breastmilk only:C-section
Breastmilk and formula:C-section
Breastmilk only:No antibiotic in labour
Breastmilk and formula:No antibiotic in labour
1m:No antibiotic in labour
1m:Breastmilk and formula
1m:Breastmilk only
1m:C-section
Breastmilk only
C-section
1m:Breastmilk and formula:No antibiotic in labour
Breastmilk and formula
No antibiotic in labour
1m:Breastmilk and formula:C-section
1m:Breastmilk only:No antibiotic in labour
1m:Breastmilk only:C-section
1m
Variables
Estimate

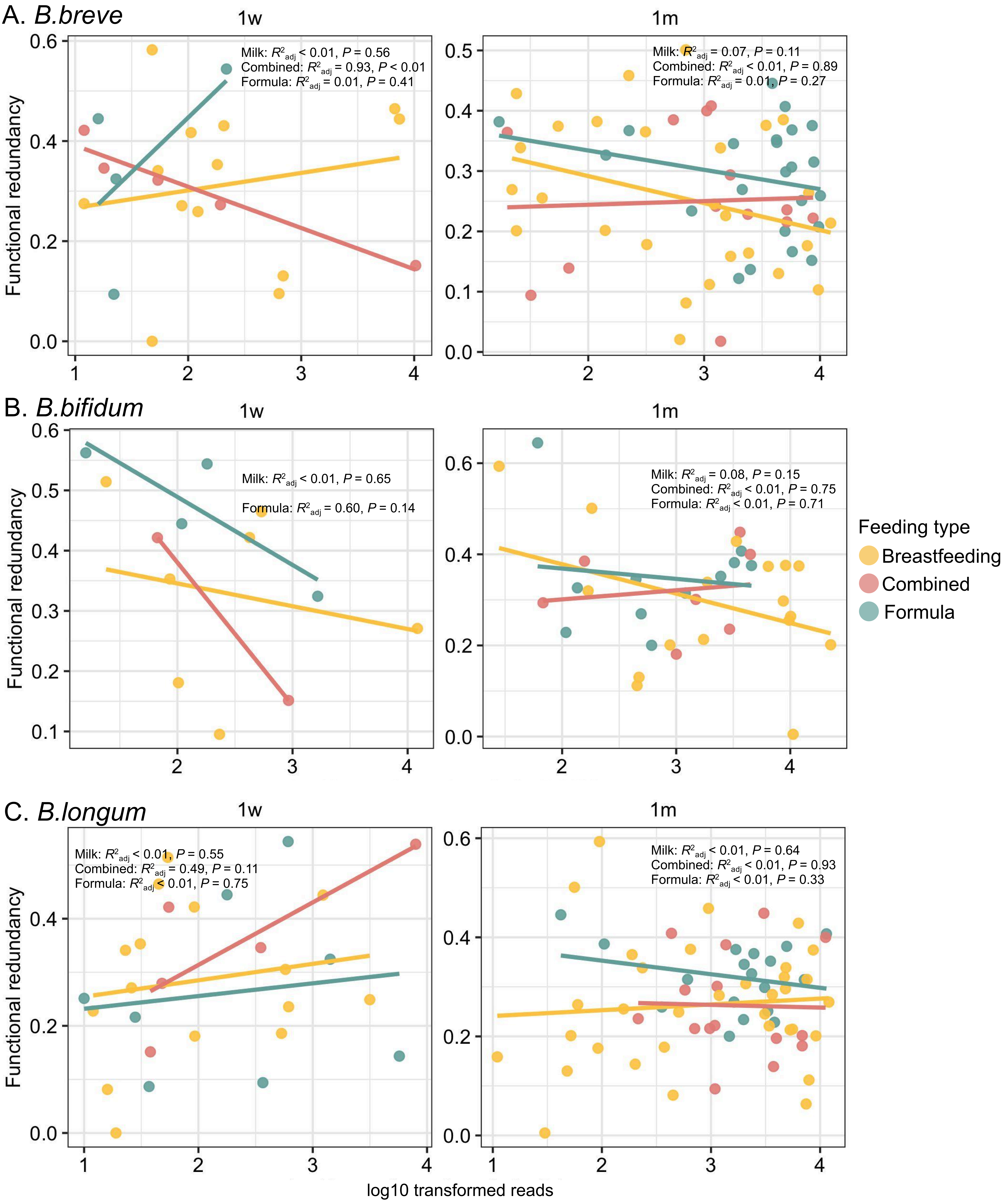

A. B.breve
1w
1m
Milk: R^2_{adj} < 0.01, P = 0.56
Combined: R^2_{adj} = 0.93, P < 0.01
Formula: R^2_{adj} = 0.01, P = 0.41
Milk: R^2_{adj} = 0.07, P = 0.11
Combined: R^2_{adj} < 0.01, P = 0.89
Formula: R^2_{adj} = 0.01, P = 0.27
B. B.bifidum
1w
1m
Milk: R^2_{adj} < 0.01, P = 0.65
Formula: R^2_{adj} = 0.60, P = 0.14
Milk: R^2_{adj} = 0.08, P = 0.15
Combined: R^2_{adj} < 0.01, P = 0.75
Formula: R^2_{adj} < 0.01, P = 0.71
C. B.longum
1w
1m
Milk: R^2_{adj} < 0.01, P = 0.55
Combined: R^2_{adj} = 0.49, P = 0.11
Formula: R^2_{adj} < 0.01, P = 0.75
Milk: R^2_{adj} < 0.01, P = 0.64
Combined: R^2_{adj} < 0.01, P = 0.93
Formula: R^2_{adj} < 0.01, P = 0.33
Functional redundancy
log10 transformed reads
Feeding type
Breastfeeding
Combined
Formula

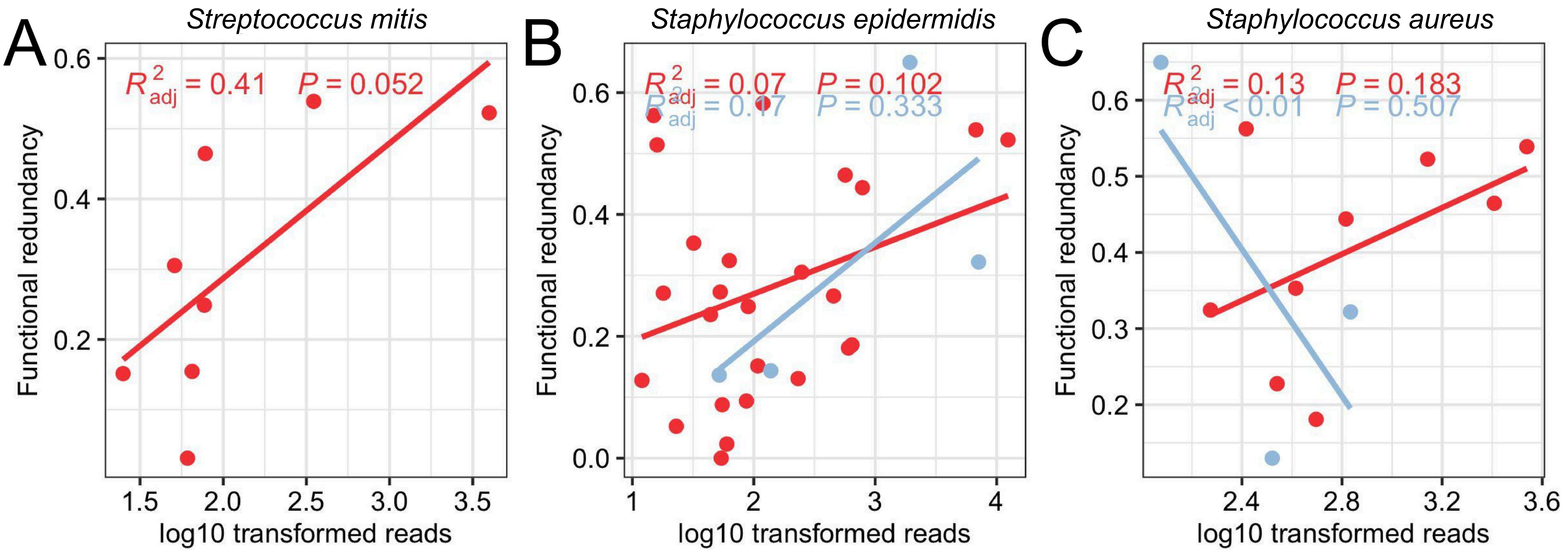

A
Streptococcus mitis
R^2_{adj} = 0.41 P = 0.052
Functional redundancy
log10 transformed reads
B
Staphylococcus epidermidis
R^2_{adj} = 0.07 P = 0.102
R^2_{adj} = 0.17 P = 0.333
Functional redundancy
log10 transformed reads
C
Staphylococcus aureus
R^2_{adj} = 0.13 P = 0.183
R^2_{adj} < 0.01 P = 0.507
Functional redundancy
log10 transformed reads

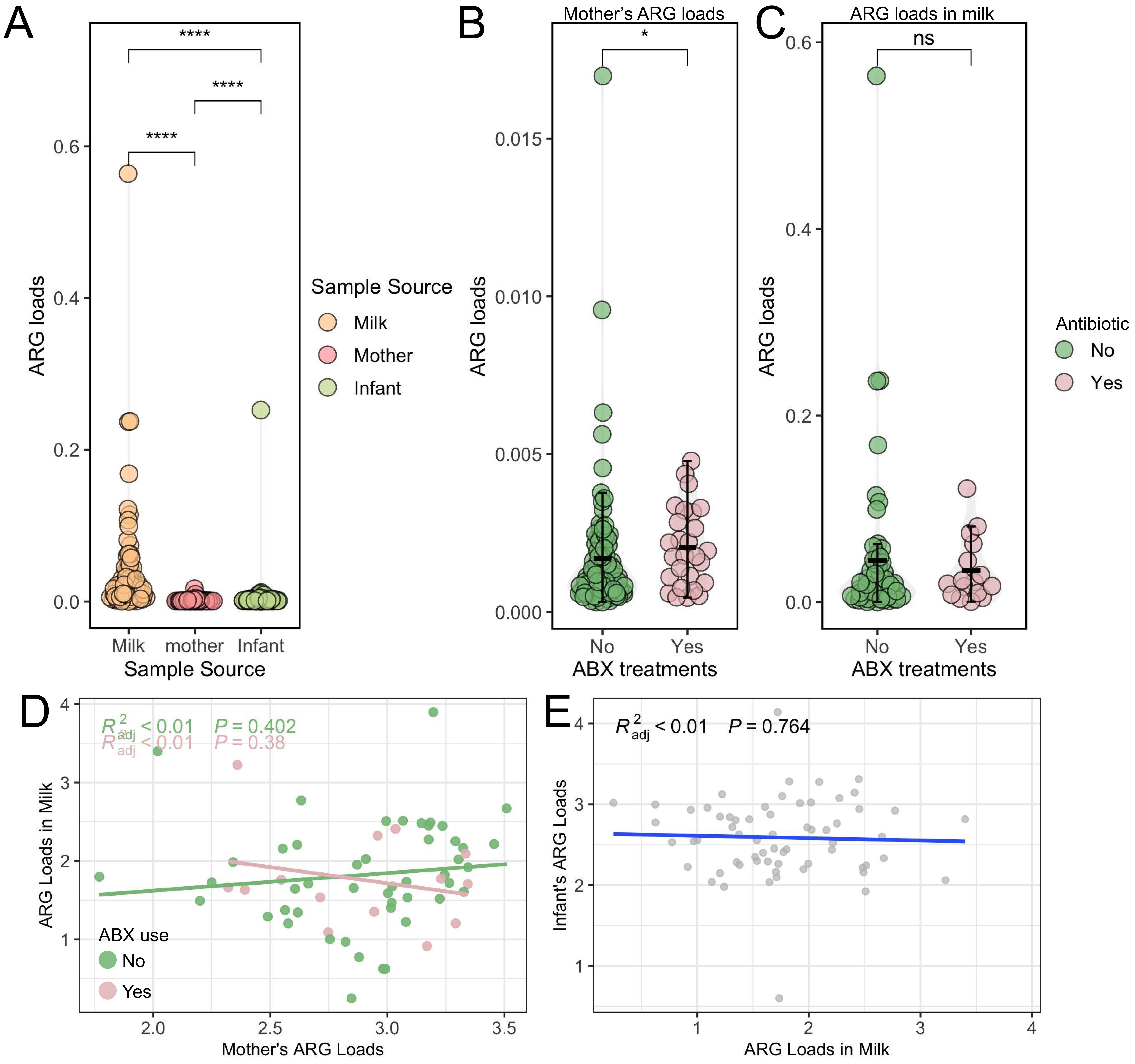
A
B
C
D
E
Sample Source
Milk
mother
Infant
Mother
ARG loads
Mother's ARG loads
ARG loads in milk
ABX treatments
No
Yes
ns
Antibiotic
$R^2_{adj} < 0.01$ $P = 0.402$
$R^2_{adj} < 0.01$ $P = 0.38$
ABX use
ARG Loads in Milk
Mother's ARG Loads
$R^2_{adj} < 0.01$ $P = 0.764$
Infant's ARG Loads

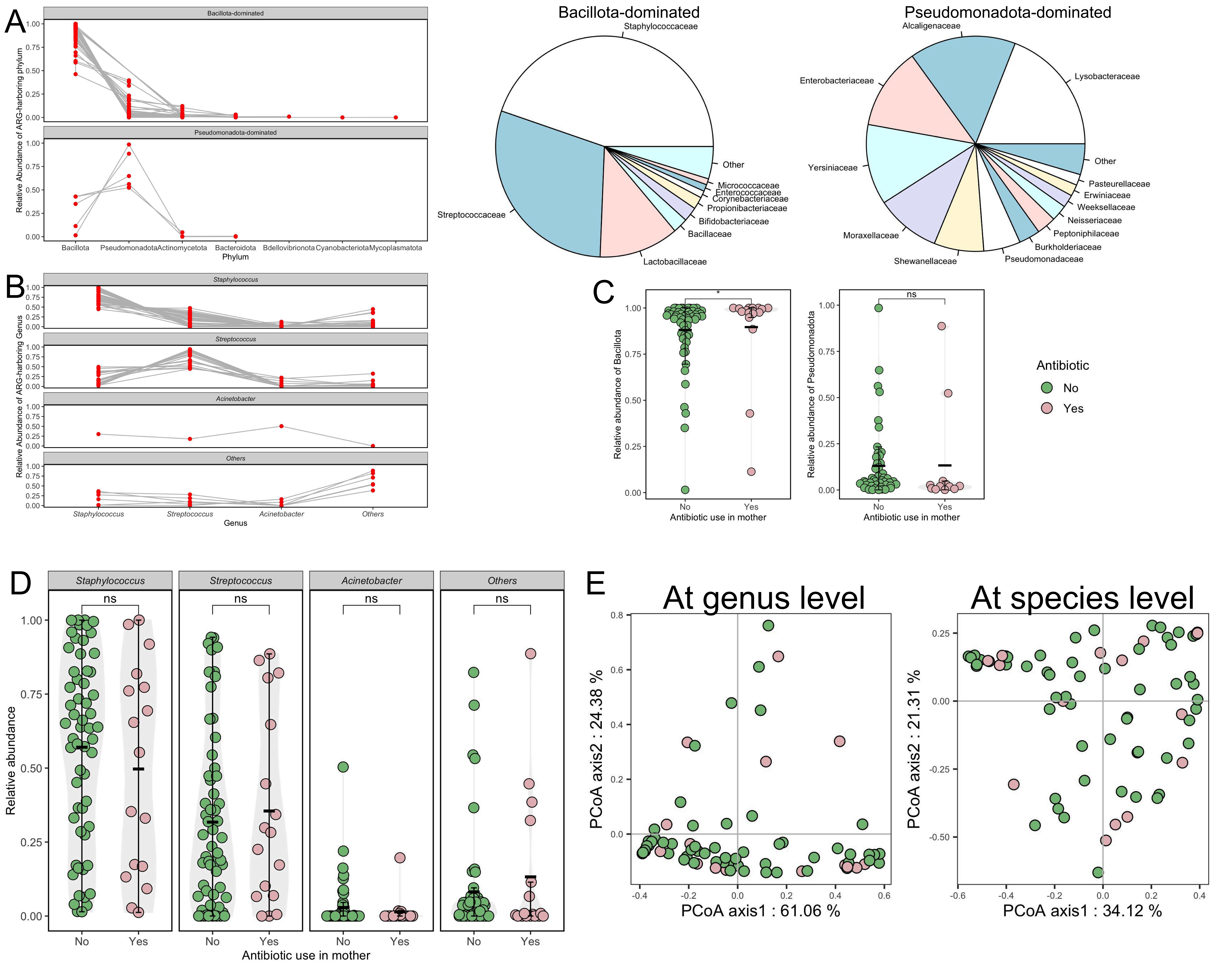

A
Bacillota-dominated
Pseudomonadota-dominated
Relative Abundance of ARG-harboring phylum
Bacillota
Pseudomonadota
Actinomycetota
Bacteroidota
Bdellovibrionota
Cyanobacteriota
Mycoplasmatota
Phylum
Bacillota-dominated
Staphylococcaceae
Other
Micrococcaceae
Enterococcaceae
Corynebacteriaceae
Propionibacteriaceae
Bifidobacteriaceae
Bacillaceae
Lactobacillaceae
Streptococcaceae
Pseudomonadota-dominated
Alcaligenaceae
Lysobacteraceae
Enterobacteriaceae
Yersiniaceae
Other
Pasteurellaceae
Erwiniaceae
Weeksellaceae
Neisseriaceae
Peptoniphilaceae
Burkholderiaceae
Pseudomonadaceae
Shewanellaceae
Moraxellaceae
B
Staphylococcus
Streptococcus
Acinetobacter
Others
Relative Abundance of ARG-harboring Genus
Genus
C
Relative abundance of Bacillota
Relative abundance of Pseudomonadota
ns
No
Yes
Antibiotic use in mother
Antibiotic
No
Yes
D
Staphylococcus
Streptococcus
Acinetobacter
Others
ns
Relative abundance
Antibiotic use in mother
E
At genus level
At species level
PCoA axis2 : 24.38 %
PCoA axis1 : 61.06 %
PCoA axis2 : 21.31 %
PCoA axis1 : 34.12 %

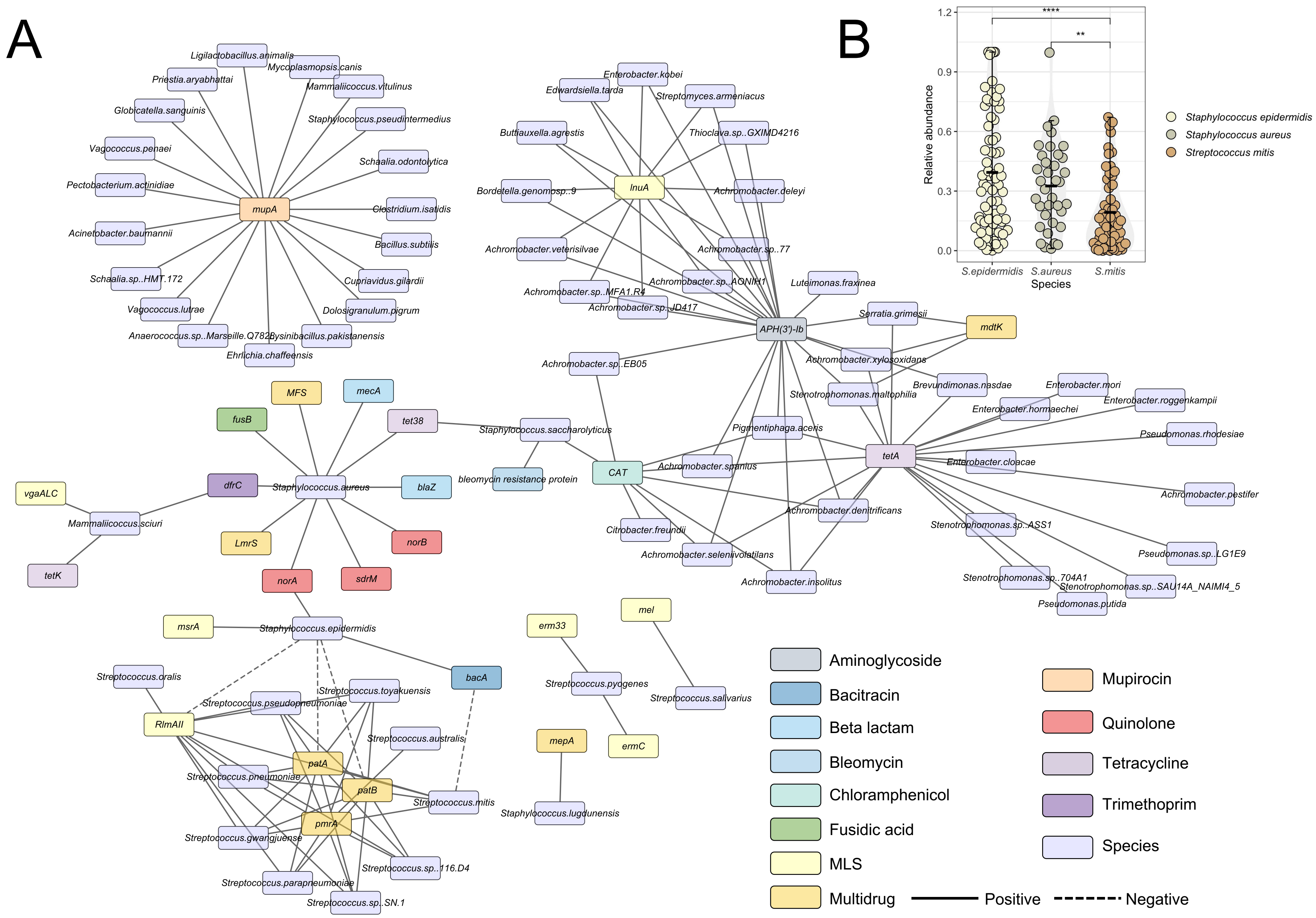
A
B
Ligilactobacillus.animalis
Mycoplasmopsis.canis
Priestia.aryabhattai
Mammaliicoccus.vitulinus
Globicatella.sanguinis
Staphylococcus.pseudintermedius
Vagococcus.penaei
Schaalia.odontolytica
Pectobacterium.actinidiae
mupA
Clostridium.isatidis
Acinetobacter.baumannii
Bacillus.subtilis
Schaalia.sp..HMT.172
Cupriavidus.gilardii
Vagococcus.lutrae
Dolosigranulum.pigrum
Anaerococcus.sp..Marseille.Q7828
Lysinibacillus.pakistanensis
Ehrlichia.chaffeensis
Enterobacter.kobei
Edwardsiella.tarda
Streptomyces.armeniacus
Buttiauxella.agrestis
Thioclava.sp..GXIMD4216
Bordetella.genomosp..9
lnuA
Achromobacter.deleyi
Achromobacter.veterisilvae
Achromobacter.sp..77
Achromobacter.sp..AONIH1
Luteimonas.fraxinea
Achromobacter.sp..MFA1.R4
Achromobacter.sp..JD417
Serratia.grimesii
APH(3')-Ib
mdtK
Achromobacter.sp..EB05
Achromobacter.xylosoxidans
Brevundimonas.nasdae
Enterobacter.mori
Stenotrophomonas.maltophilia
Enterobacter.roggenkampii
Enterobacter.hormaechei
Pigmentiphaga.aceris
Pseudomonas.rhodesiae
tetA
Achromobacter.spanius
Enterobacter.cloacae
CAT
Achromobacter.pestifer
Achromobacter.denitrificans
Stenotrophomonas.sp..ASS1
Citrobacter.freundii
Achromobacter.seleniivolatilans
Pseudomonas.sp..LG1E9
Achromobacter.insolitus
Stenotrophomonas.sp..704A1
Stenotrophomonas.sp..SAU14A_NAIMI4_5
Pseudomonas.putida
MFS
mecA
fusB
tet38
Staphylococcus.saccharolyticus
bleomycin resistance protein
dfrC
Staphylococcus.aureus
blaZ
vgaALC
Mammaliicoccus.sciuri
LmrS
norB
tetK
norA
sdrM
msrA
Staphylococcus.epidermidis
erm33
mel
Streptococcus.oralis
bacA
Streptococcus.pyogenes
Streptococcus.salivarius
Streptococcus.toyakuensis
Streptococcus.pseudopneumoniae
RlmAII
Streptococcus.australis
mepA
ermC
patA
Streptococcus.pneumoniae
patB
Streptococcus.mitis
Staphylococcus.lugdunensis
pmrA
Streptococcus.gwangjuense
Streptococcus.sp..116.D4
Streptococcus.parapneumoniae
Streptococcus.sp..SN.1
Aminoglycoside
Bacitracin
Beta lactam
Bleomycin
Chloramphenicol
Fusidic acid
MLS
Multidrug
Mupirocin
Quinolone
Tetracycline
Trimethoprim
Species
Positive
Negative
Relative abundance
1.2
0.9
0.6
0.3
0.0
****
**
S.epidermidis
S.aureus
S.mitis
Species
Staphylococcus epidermidis
Staphylococcus aureus
Streptococcus mitis

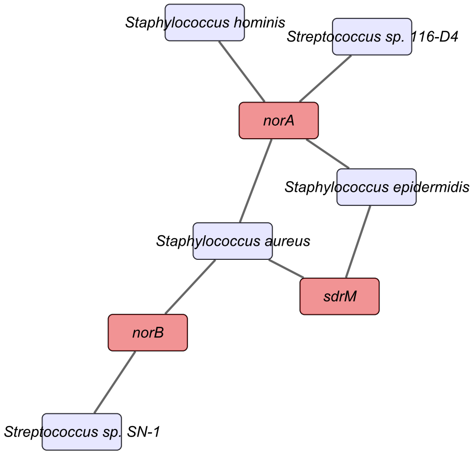
Staphylococcus hominis
Streptococcus sp. 116-D4
norA
Staphylococcus epidermidis
Staphylococcus aureus
sdrM
norB
Streptococcus sp. SN-1
